# MRI-measured periprostatic adipose tissue volume as a prognostic predictor in prostate cancer patients undergoing laparoscopic radical prostatectomy

**DOI:** 10.1080/21623945.2023.2201964

**Published:** 2023-04-25

**Authors:** Tianyu Xiong, Fang Cao, Guangyi Zhu, Xiaobo Ye, Yun Cui, Nianzeng Xing, Huibo Zhang, Yinong Niu

**Affiliations:** a Department of Urology, Beijing Chaoyang Hospital, Capital Medical University, Beijing, China; b Department of Urology, Beijing Shijitan Hospital, Capital Medical University, Beijing, China; c Department of Urology, Cancer Hospital Chinese Academy of Medical Sciences, Beijing, China; d Department of Radiology, Beijing Chaoyang Hospital, Capital Medical University, Beijing, China

**Keywords:** Adipose tissue, magnetic resonance imaging, prostate cancer, laparoscopic radical prostatectomy, biochemical recurrence

## Abstract

In this study, we evaluated the association between the PPAT volume and the prognosis of PCa patients after LRP. We retrospectively analysed data of 189 PCa patients who underwent LRP in Beijing Chaoyang Hospital. Volumes of PPAT and prostate were measured by magnetic resonance imaging (MRI), and normalized PPAT volume was computed (PPAT volume divided by prostate volume). Patients were then stratified into the high-PPAT group (*n* = 95) and low-PPAT group (*n* = 94) by the median of normalized PPAT volume (73%). The high-PPAT group had significantly higher Gleason score (total score 8 or more, 39.0% vs. 4.3%, *p* < 0.001) and pathological stage (stage T3b, 28.4% vs. 13.8%, *p* = 0.048). No significant correlation between normalized PPAT volume and body mass index (ρ = −0.012, *p* = 0.872) was observed. Kaplan-Meier curve analysis showed the high-PPAT group had significantly shorter biochemical recurrence (BCR) interval (median progression-free survival time 15.9 months vs. 32.7 months, *p* = 0.001). Univiarate and multivariate Cox regression analyses showed high normalized PPAT volume (>73%) (hazard ratio 1.787 [1.075–3.156], *p* = 0.002) were independent risk factors for BCR post-operatively. In conclusion, MRI-measured PPAT volume is of significant prognostic value for PCa patients undergoing LRP.

## Introduction

The relationship between obesity and poor treatment outcome has been reported in multiple types of cancer [1,2,3]. Obesity is a risk factor for poor treatment outcome and increased cancer-specific mortality in different types of cancer, including oesophageal, gynaecological and colorectal cancers. Several recent studies also revealed an association between obesity and aggressiveness of prostate cancer (PCa), including an increased risk of biochemical recurrence (BCR) following radical prostatectomy (RP) and higher mortality [4–6], whereas the relationship between obesity and clinical outcomes of PCa was still inconclusive [7–9]. These controversial results may be due to the fact that body mass index (BMI), which is the most commonly used indicator for obesity, provides no insight into the distribution of adipose tissue. The understanding of the impact of adipose tissue on the development of PCa was still limited.

Periprostatic adipose tissue (PPAT), the adipose tissue surrounding the prostate gland, has been reported to play an important role in PCa progression [10]. Previous studies suggested PPAT represented a distinct variable for PCa patients rather than a surrogate parameter for general body mass. The PPAT amount was significantly correlated with higher Gleason score and advanced tumour stage, reflecting a trend with more aggressive cancer [11–15]. High PPAT volume measured on computed tomography (CT) or magnetic resonance imaging (MRI) was found to be a predictor for poor prognosis after treatment, such as shorter progression-free survival and overall survival after receiving androgen deprivation therapy (ADT) and radiotherapy [16–19]. However, the association between PPAT amount and prognosis of patients who underwent RP still remains unclear. In this study, we aimed to investigate whether MRI-measured PPAT volume was an independent prognostic factor for PCa patients who underwent laparoscopic radical prostatectomy (LRP) and assess its predictive value for BCR post-operatively.

## Methods

### Patient selection

In this cross-sectional study, we retrospectively analysed the data of patients with PCa who underwent LRP from July 2010 to August 2021 in Beijing Chaoyang Hospital, Capital Medical University. All patients were diagnosed with PCa by prostate biopsy or specimens of transurethral resection of prostate (TURP) and were routinely evaluated by prostate MRI and serum prostate-specific antigen (PSA) level before prostate biopsy or TURP. LRP was performed through extraperitoneal approach by two urology surgeons with at least 5-year experience in laparoscopic surgery. Patients who had no available prostate MRI images or clinicopathologic information prior to biopsy or TURP were excluded. In addition, we also excluded the patients who were diagnosed of bone metastasis or received any kind of neoadjuvant therapy prior to LRP. Our study was conducted in accordance with the Declaration of Helsinki (as revised in 2013) and was approved by the Institutional Review Board of Beijing Chaoyang Hospital, Capital Medical University (NO.: 2022-Ke-55), which waived the requirement of informed consent for this retrospective analysis.

### Data collection

Clinicopathologic parameters including age, BMI, serum PSA level at initial diagnosis, Gleason scores of LRP specimens and pathological T stage were collected retrospectively by reviewing medical records. Data of BCR were collected during outpatient follow-up. BCR was defined as two consecutive PSA values≥0.2 ng/mL after LRP. If the post-operative PSA levels failed to decrease below 0.2 ng/mL, the date of RP was defined as the date of BCR. The biochemical recurrence free survival (BFS) time was calculated from the date of surgery to the date of BCR or the last follow-up visit alternatively. Patients without BCR were censored at the last follow-up visit.

### PPAT measurement

**Figure 1.**
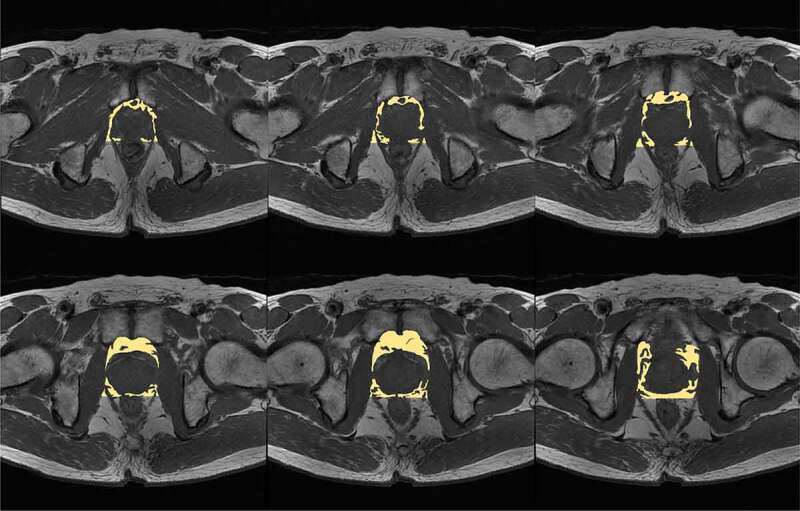
Measurement of PPAT volume on multiple T1-weighted slices. PPAT: periprostatic adipose tissue.

All patients in this study received 3.0T MRI examination at our hospital prior to prostate biopsy or TURP. All the images were taken within three months prior to LRP. MRI was performed with a 3.0-Tesla scanner with a pelvic phased-array surface coil without an endorectal coil. PPAT volume was defined as the adipose tissue surrounding the prostate and anterior to the rectum, which was outlined including the first visible fascial boundary adjacent to the levator muscles laterally, Denonvilliers fascia posteriorly, and the pubic symphysis anteriorly. We measured PPAT volume on multiple transverse T1-weighted planes from multiple sequential slices starting from apex to base of prostates (Figure 1) and was calculated by employing the formula where volume = the sum of contour area × slice thickness. PPAT measurement was performed manually using ImageJ software (version 1.53). All the measurement procedures were performed by two urologists who knew that all males had undergone prostate biopsy but were blinded to the clinical and pathological information. The average of the results measured by the two urologists was used in the analysis. Prostate volume was measured and calculated by the same method. The inter-observer reliability was tested using intraclass correlation coefficient (ICC). To minimize the effect of prostate volume on PPAT volume, we divided PPAT volume by the prostate volume to compute the normalized PPAT volume following the method of previous research [20]. The normalized PPAT volume was expressed as percentage in the analysis.

### Statistical analysis

In our study, normally distributed continuous variables were expressed as mean values with standard deviations and were compared using student t-test. Non-normally distributed continuous variables were expressed as median and interquartile ranges and were compared using the Mann-Whitney test. Categorical variables were compared using the chi-square test or Fisher’s exact test as appropriate. Spearman correlation coefficient (ρ) was used to evaluate the relationship between PPAT amount and clinical parameters. The correlation between the PPAT volume and BFS after LRP was analysed using the Kaplan-Meier method. Univariate and multivariate Cox regression analyses were used to calculate the respective hazard ratio and 95% confidence intervals for the PPAT volume and other clinical and pathological factors. Variables with a *p-*value<0.1 in univariate analysis were entered into the multivariate analysis. SPSS version 26.0 (IBM Corp., Armonk, NY, USA) and R version 4.1.2 were utilized for statistical analysis. A *p*-value<0.05 was considered significant for all parameters.

## Results

A total of 517 consecutive patients were included in the present study. Patients missing MRI scans and other clinical data (*n* = 242) were excluded. Patients with bone metastasis or those who were received ADT (*n* = 86) were also excluded, resulting in a cohort of 189 patients. No patient died before BCR was observed in this cohort. The baseline clinical characteristics and PPAT measurement results of all patients were presented in Table 1. The median age and BMI was 68 years and 25.57 kg/m^2^, respectively. A total of 104 (55.0%) men in our study were overweight (BMI≥25 kg/m^2^) and 12 (6.3%) were obese (BMI≥30 kg/m^2^). The median preoperative serum PSA level was 12.52 ng/mL. Forty-one (21.7%) patients had a total Gleason score of 8 or more in LRP specimens. Sixty-six (34.9%) patients had pT3a/b stage. The median PPAT and prostate volumes were 29.94 and 38.35 cm^3^, respectively. Excellent reproducibility of measurement was determined using ICC and assessed as 0.986 for PPAT (*p* < 0.001) and 0.975 for subcutaneous fat thicknesses (*p* < 0.001).

**Table 1.** Clinical characteristics and adipose features of patients by PPAT amount. *Compared between the High-PPAT group and the Low-PPAT group. IQR: interquartile range; SD: standard deviation; BMI: body mass index; PSA: prostate-specific antigen; PPAT: periprostatic adipose tissue.

Variables	Total (*n* = 189)	High-PPAT group (*n* = 95)	Low-PPAT group (*n* = 94)	*p* value*
Age (year), median (IRQ)	68 (64–73)	68 (65–73)	67 (63–73)	0.109
BMI (kg/m^2^), mean ± SD	25.57 ± 2.98	25.48 ± 2.89	25.66 ± 3.08	0.673
PSA (ng/mL), median (IRQ)	12.52 (8.28–22.16)	12.61 (7.93–25.25)	12.22 (8.57–19.20)	0.510
Prostate volume (cm^3^), median (IRQ)	38.35 (30.48–53.48)	27.71 (33.90–44.89)	44.57 (36.56–62.13)	<0.001
PPAT volume(cm^3^), median (IRQ)	29.94 (23.37–38.48)	31.43 (26.09–40.29)	27.69 (21.36–34.14)	0.002
Normalized PPAT volume (%), median (IRQ)	73.00 (61.01–90.72)	90.66 (80.57–105.08)	61.01 (50.40–68.09)	<0.001
Gleason score group, n (%)				<0.001
6	34 (18.0)	14 (14.7)	20 (21.3)	
3 + 4	66 (34.9)	26 (27.4)	40 (42.5)	
4 + 3	48 (25.4)	18 (18.9)	30 (31.9)	
8	12 (6.4)	11 (11.6)	1 (1.1)	
9 or 10	29 (15.3)	26 (27.4)	3 (3.2)	
Pathological T stage, n (%)				0.044
T2	123 (65.1)	55 (57.9)	68 (72.4)	
T3a	26 (13.8)	13 (13.7)	13 (13.8)	
T3b	40 (21.1)	27 (28.4)	13 (13.8)	
Positive surgical margin, n (%)	96 (49.2)	54 (56.8)	42 (44.7)	0.095

We used the median of normalized PPAT volume (73%), which meant PPAT volume was 73% of the prostate volume, to stratify patients into the high-PPAT group (normalized PPAT volume≥73%, *n* = 95) and low-PPAT group (normalized PPAT volume<73%, *n* = 94). Patients in the high-PPAT group had significantly higher Gleason scores (total score 8 or more, 39.0% *vs*. 4.3%, *p* < 0.001) and pathological stage (stage T3b, 28.4% *vs*. 13.8%, *p* = 0.048). No significant difference was observed in BMI between the two group (25.48 ± 2.89 *vs*. 25.66 ± 3.08, *p* = 0.673). In Spearman correlation analysis, normalized PPAT volume was significantly correlated with age (ρ = 0.144, *p* = 0.048). Meanwhile, no significant correlation between normalized PPAT volume and BMI (ρ = −0.012, *p* = 0.872) was observed (Table 2).

**Table 2.** The relationship between normalized PPAT volume, age, BMI and serum PSA level. BMI: body mass index; PSA: prostate-specific antigen; PPAT: periprostatic adipose tissue.

		Normalized PPAT volume	Age	BMI	Serum PSA level
Normalized PPAT volume	Spearman correlation coefficient (ρ)	1	0.144	−0.012	0.008
	Sig. (two-tailed)	.	0.048	0.872	0.914
Age	Spearman correlation coefficient (ρ)	0.144	1	−0.109	0.018
	Sig. (two-tailed)	0.048	.	0.137	0.801
BMI	Spearman correlation coefficient (ρ)	−0.012	−0.109	1	0.078
	Sig. (two-tailed)	0.872	0.137	.	0.287
Serum PSA level	Spearman correlation coefficient (ρ)	0.008	0.018	0.078	1
	Sig. (two-tailed)	0.914	0.801	0.287	.

**Figure 2.**
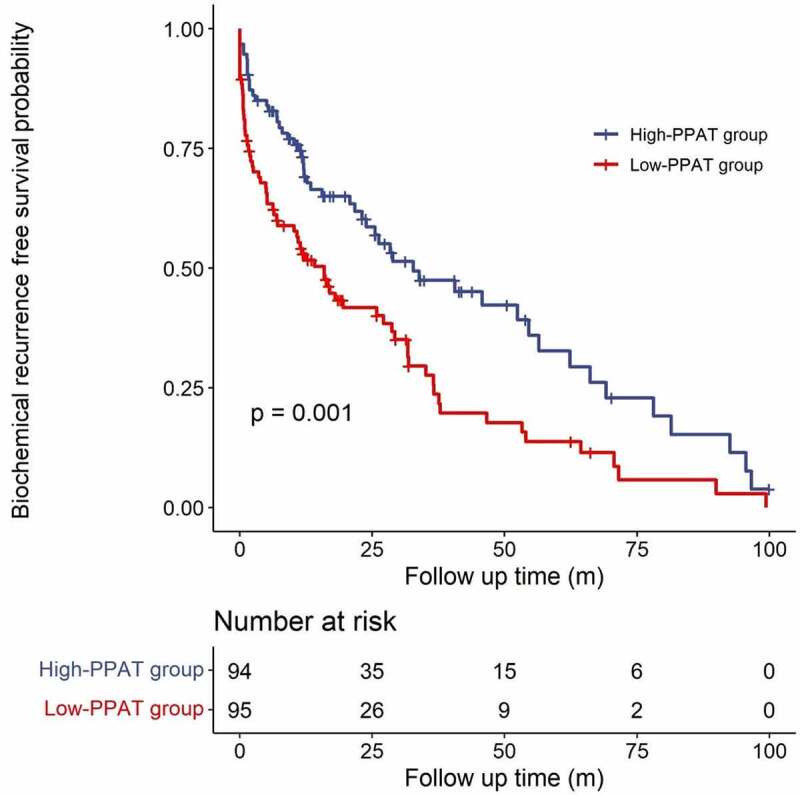
Kaplan-Meier curve analysis of biochemical recurrence free survival of patients by normalized PPAT volume after laparoscopic radical prostatectomy. PPAT: periprostatic adipose tissue.

The median follow-up period of all patients was 12.1 months (interquartile range 3.2–30.4). A total of 124 (65.6%) patients reported developed BCR post-operatively. Among them, 13 (6.9%) patients failed to show a <0.2 ng/mL PSA level post-operatively. Seventy-one (74.7%) patients in the high-PPAT group and 53 (56.3%) in the low-PPAT group developed BCR after LRP. The median BFS times for patients in the high-PPAT group and low-PPAT group was 15.9 and 32.7 months, respectively, and Kaplan-Meier curve analysis showed that patients in the high-PPAT group had significantly shorter BFS (*p* = 0.001), as shown in Figure 2. Univiarate and multivariate Cox regression analyses showed high normalized PPAT volume (>73%) (hazard ratio 1.787 [1.075–3.156], *p* = 0.002), serum PSA level>10 ng/mL (hazard ratio 1.625 [1.113–2.372], *p* = 0.012), pathological Gleason score≥8 (hazard ratio 1.466 [1.005–2.140], *p* = 0.047) and positive surgical margin (hazard ratio 1.762 [1.213–2.561], *p* = 0.003) were independent risk factors for BCR after LRP (Table 3).

**Table 3.** Univariate and multivariate Cox regression analyses for prediction of biological recurrence. HR: hazard ratio; CI: confidence interval; BMI: body mass index; PSA: prostate-specific antigen; PPAT: periprostatic adipose tissue.

	Univariate analysis		Multivariate analysis	
Variables	HR (95% CI)	*p* value	HR (95% CI)	*p* value
High normalized PPAT volume (>73%)	1.791 (1.252–2.562)	0.001	1.787 (1.075–3.156)	0.002
Age>70 years	1.234 (0.859–1.772)	0.255		
BMI>25 kg/m^2^	0.859 (0.601–1.229)	0.406		
PSA>10 ng/mL	1.639 (1.129–2.378)	0.009	1.625 (1.113–2.372)	0.012
Pathological Gleason score≥8	1.761 (1.221–2.542)	0.002	1.466 (1.005–2.140)	0.047
Pathological T3 stage	1.700 (1.178–2.452)	0.005	1.227 (0.822–1.832)	0.317
Positive surgical margin	1.841 (1.275–2.659)	0.001	1.762 (1.213–2.561)	0.003

## Discussion

Despite the poor characterization and definition, PPAT has been recently emerged as a potential factor in the development of PCa. PPAT surrounds the prostate gland and is separated by a fibromuscular capsule [21]. Although located in the pelvic region, PPAT is often considered as visceral adipose tissue because of its distinct biological nature [22–24]. Unlike other adipose depots, PPAT does not increase in volume in obese patients, and no elevated levels of chronic inflammation and fibrosis were observed in PPAT [24]. PPAT secrets multiple kinds of cytokine, including IL-6, TNF-ɑ and MMP9, which participates in the development of tumour growth and invasiveness [22–24]. The chemokine CCL7 secreted by PPAT was reported to mediate interactions between PPAT and PCa cells through the CCR3/CCL7 axis, promoting the extraprostatic extension of tumour [25]. These findings suggest that PPAT is a metabolically active endocrine organ and may affect tumour progression in prostate. Several studies had also reported the correlation between imaging features of PPAT and PCa aggressiveness [12,16,17,19]. van Roermund *et al* first reported the CT-measured PPAT amount was associated with Gleason score≥8 cancer in prostate biopsy [12]. Salji *et al* and Huang et al found that PPAT volume was a predictor for time to castration-resistant prostate cancer (CRPC) in patients receiving ADT, reflecting poor tumour response to hormone treatment [16,17]. For patients who received radiation therapy, Di Bella *et al* reported that PPAT abundance was associated with increased risk of recurrence [19]. These results indicated that PPAT had a close relationship with more aggressive PCa and poor treatment outcome. On the other hand, the understanding of the effect of PPAT amount in patients who underwent RP is still limited. Dahran *et al* analysed the 162 patients with localized PCa who underwent RP and reported that patients with post-operative Gleason score 7 or more had higher normalized PPAT volume, suggesting the close relationship between PPAT and PCa aggressiveness [26]. However, the impact of PPAT amount on the long-term prognosis in patients after RP treatment was not yet fully revealed.

In this study, we analysed the predictive value of PPAT amount for poor prognosis of PCa patients who underwent LRP. We measured the PPAT volume by MRI (Figure 1) and the definition standards were in accordance with the previous report [20]. To provide a better reflection of relative fat volume in the pelvis and periprostatic area, the PPAT volume was normalized by prostate volume to create the normalized PPAT volume. No significant difference was observed in BMI between the two groups (Table 1), and normalized front PPAT volume were not significantly correlated with BMI (Table 2), suggesting PPAT amount was not affected by the overall obesity degree. Patients in the high-PPAT group had higher Gleason score and pathological T stage (Table 1). Furthermore, high normalized PPAT volume (>73%) was significantly correlated with shorter BFS (Figure 2) and was an independent risk factor for BCR post-operatively (Table 3). To our knowledge, this is the first study which analysed the predictive value of PPAT volume for poor prognosis in PCa patients who underwent LRP.

There were several limitations in our study. First, it is a single-centre retrospective study, resulting in the potential selection bias. Second, due to the technical limitation, we only analysed the spatial features of PPAT. The underlying mechanism of biological recurrence driven by PPAT is need to be explored in the future. Third, the measurement of PPAT volume in our study was time consuming and the calculation method needs to be improved.

In conclusion, we analysed the prognostic value of normalized PPAT volume, a derivative index normalized by prostate volume, in a cohort of PCa patients undergoing LRP. We found that the high normalized PPAT volume was correlated with high grade PCa and high T stage and was an independent risk factor for BCR post-operatively. Our work suggests that imaging features of PPAT may have important implication for deep understanding of PCa development and further improvement of the management of PCa.

## Data Availability

The data that support the findings of this study are available on request from the corresponding author, Yinong Niu. The data are not publicly available due to restriction by the Institutional Review Board of Beijing Chaoyang Hospital, Capital Medical University, in order to protect the patient privacy.
